# Structural synaptic plasticity across sleep and wake

**DOI:** 10.1016/j.cophys.2019.12.007

**Published:** 2020-06

**Authors:** Michele Bellesi, Luisa de Vivo

**Affiliations:** School of Physiology, Pharmacology and Neuroscience, University of Bristol, Biomedical Sciences Building, University Walk, BS8 1TD Bristol, UK

## Abstract

Sleep-dependent synaptic plasticity is crucial for optimal cognition. However, establishing the direction of synaptic plasticity during sleep has been particularly challenging since data in support of both synaptic potentiation and depotentiation have been reported. This review focuses on structural synaptic plasticity across sleep and wake and summarizes recent developments in the use of 3-dimensional electron microscopy as applied to this field.

**Current Opinion in Physiology** 2020, **15**:74–81

This review comes from a themed issue on **Physiology of sleep**

Edited by **Vladyslav Vyazovskiy** and **Jenny Morton**

For a complete overview see the Issue and the Editorial

Available online 23rd December 2019

**https://doi.org/10.1016/j.cophys.2019.12.007**

2468-8673/© 2020 The Authors. Published by Elsevier Ltd. This is an open access article under the CC BY license (http://creativecommons.org/licenses/by/4.0/).

## Introduction

Why animals sleep is one of the most interesting questions in biology, and despite the significant progresses made in identifying the brain circuits that regulate sleep in different species, the ultimate answer to why sleep has evolved remains elusive. Sleep is particularly important for the brain where it ensures optimal biological functioning by serving restorative and detoxification purposes [1,2] and, enhances cognitive and complex motor tasks [3, 4, 5, 6, 7]. Sleep’s beneficial role for cognition is likely to be carried out by synaptic plasticity, although other processes promoted by sleep (e.g. waste clearance, restoration of energy or cellular components) may also play a role. Synaptic potentiation and/or depotentiation (i.e. synaptic plasticity) are the primary mechanisms of skill learning and improving behavioural performance. However, the direction of synaptic plasticity during sleep is a controversial topic since evidence supporting both synaptic potentiation and depotentiation have been gathered by different groups, at the molecular and electrophysiological levels (reviewed in Refs. [8, 9, 10]).

Structural synaptic plasticity across sleep and wake was initially studied in fruit flies by measuring protein levels, density and size of synaptic structures using confocal microscopy [11, 12, 13]. These studies showed that sleep was associated with decreased density or volume of fluorescent pre-synaptic and post-synaptic markers (e.g. Bruchpilot, disk-large, synaptobrevin) and with lower dendrite length and branching relative to waking and sleep deprivation [13]. Subsequently, other studies used *in vivo* two-photon imaging to follow the dynamics of pre-synaptic and post-synaptic structures across sleep and wake in zebrafish larvae and in mice, confirming the presence of circadian and sleep/wake-dependent structural plasticity in vertebrates too [14, 15, 16]. It was found that, in the somatosensory cortex of adolescent mice, fluorescently labelled dendritic spines undergo a constant turnover and that wake is associated with a small (∼2%) but significant net increase of dendritic spine number, whereas sleep leads to a net spine loss. However, spine density was found to remain constant in the cortex of adult mice [15].

Since then, the study of sleep-dependent structural plasticity has gained much attention and multiple research groups measured spine density and turnover across sleep, wake and sleep deprivation in the hippocampus [17, 18, 19], dentate gyrus [20,21], prefrontal cortex [18,22], somatosensory and motor cortex [23,24,25^••^] finding sometimes completely opposite results. On one hand, studies that used Golgi impregnation to stain dorsal hippocampal neurons reported that 5 hours of acute sleep deprivation by gentle handling could lead to reduced spine density and size in CA1 and in the inferior blade of the dentate gyrus relative to sleep [18,19,26]. On the other hand, using fluorescent molecules to label CA1 hippocampal neurons resulted in more numerous and bigger spines after sleep deprivation relative to sleep [17,27]. Since a similar regimen of sleep deprivation was applied, the opposite findings might be explained by the fact that the Golgi method stains unpredictably only some neurons and not others, applying an unknown bias in the population of spines sampled. Hence, it is possible for the decrease in spine density after sleep deprivation to be specific for the population of neurons labelled by the Golgi staining, and therefore to be lost when other methods to visualize dendritic spines are applied. However, another study reported that 24-hour sleep deprivation led to increased spine density in the prefrontal cortex of 22-month old rats but not in 3-month old rats, and to reduced spine density in the CA1 of 3-month old rats, but not of 22-month old rats [18]. Thus, sleep-dependent synaptic plasticity can be differentially modulated by the animal’s age, the brain region considered, and the kind of sleep deprivation applied.

Time-lapse two-photon microscopy of fluorescent dendritic segments and spines have also been used to study the effect of acute sleep deprivation on spine turnover and stabilization in the mouse motor cortex during development (at postnatal day 21) and after motor skill learning [23,24]. These studies, even if they did not provide measures of total spine density across sleep and wake, suggested that sleep after training can at the same time promote pruning of some spines and be permissive of the formation or potentiation of other spines. Indeed, another study that used *in vivo* electroporation to transfect layer V pyramidal neurons of primary motor cortex with dsRed2, a structural marker, and SuperEcliptic pHluorin-tagged GluA1 (SEP-GluA1), which specifically visualizes surface inserted GluA1 AMPA receptor subunits, found that some dendritic spines became bigger and expressed more GluA1 after sleep relative to wake. However, the spines that became bigger and acquired more GluA1 subunits were only a minority since, in general, sleep was associated with an overall decrease of spine size (reduced dsRed intensity) and with synaptic depotentiation (reduced SEP-GluA1 intensity) [25^••^].

## Morphological markers of synaptic plasticity: beyond spine density

**Figure 1:**
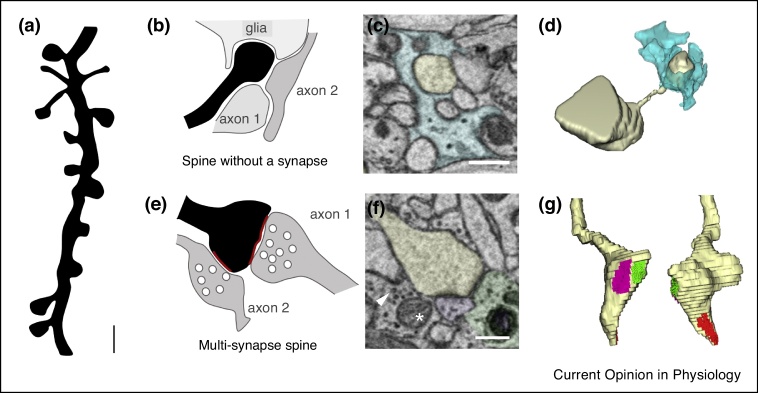
Not all spines carry one synapse. **(a)** Schematic illustration of a dendritic segment with spines of different shape and size as visualized in 2 dimensions at the light microscopy resolution. Scale bar 1 μm. **(b)** Schematic illustration of a dendritic spine (in black) lacking a synapse because surrounded by glial processes and axons (grey) without pre-synaptic vesicles. **(c)** An electron micrograph showing a dendritic spine head (in yellow) lacking a synapse and surrounded by an astrocytic process (light blue) and other structures such axons and dendrites in the mouse somatosensory cortex. Scale bar 350 nm. **(d)** 3d reconstruction of the spine shown in (f) and astrocyte showed in (c). **(e)** Schematic illustration of a dendritic spine (black) making 2 synaptic contacts with different pre-synaptic terminals (grey); **(f)** electron micrograph of a multi-synapse spine (yellow) and two out of three contacting pre-synaptic terminals (light green and purple) in the mouse somatosensory cortex. Asterisk indicates a mitochondrion; arrowhead indicates a pre-synaptic vesicle. Scale bar 300 nm. **(g)** 3d reconstruction of the spine and its 3 synaptic contacts in magenta, green, and red, front and back view.

Dendritic spines are commonly considered to be surrogate of excitatory synapses because most of them carry a single excitatory synapse [28]. Because of a poor spatial resolution especially on the z axis, quantification of spine density by using light microscopy can underestimate the presence of small spines with very thin (40−200 nm) and long (>1 μm) necks or of those that lie orthogonal to the plane of imaging. Further bias in the measure of synaptic plasticity are given by the fact that electrode implantation, skull craniotomies and thinning performed to allow EEG recordings or longitudinal *in vivo* two-photon imaging are major surgeries that can trigger inflammatory responses and glia activation and lead to subtle changes in spine turnover and fluorescent signal to noise ratio over time [29]. Moreover, relying on dendritic spines as a measure of synapse number, does not account for the existence of spines carrying multiple synapses [30, 31, 32, 33, 34] nor for spines without synaptic specializations or a functional pre-synaptic partner (e.g. spines contacting a vesicle-free pre-synaptic element) [35, 36, 37, 38], whose function and abundance in different brain regions, ages and after specific wake-dependent experiences are not known (Figure 1).

**Figure 2:**
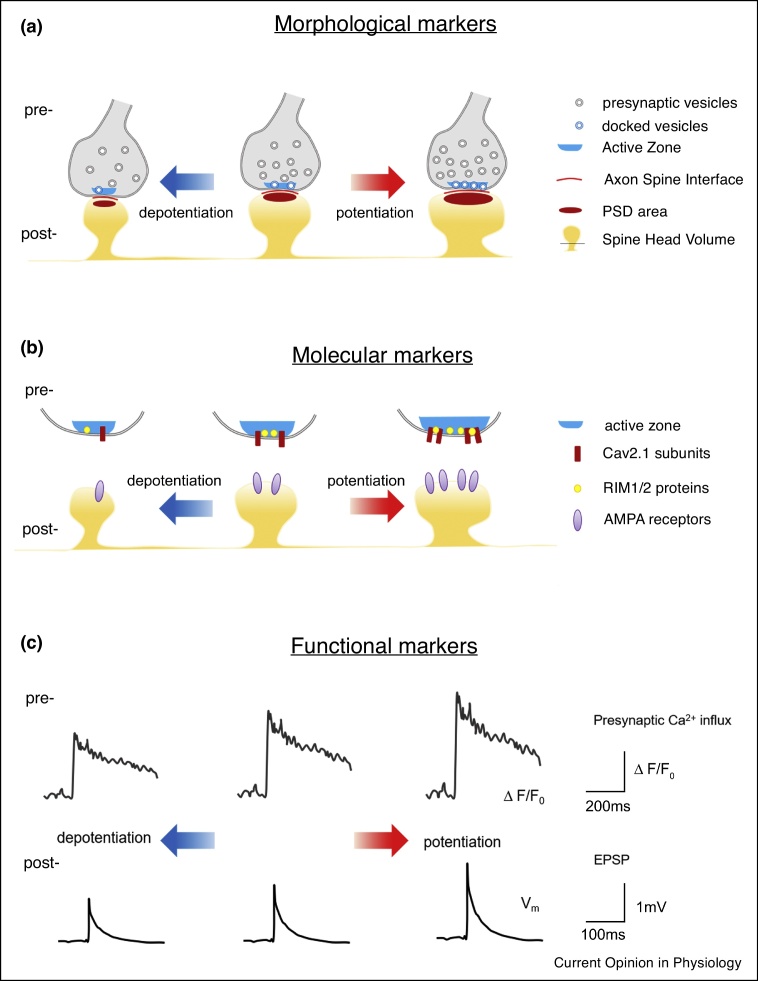
Markers of synaptic strength. Synaptic strength varies as a result of synaptic plasticity which is associated with bidirectional changes of morphological, molecular and functional parameters, at the pre-synaptic and post-synaptic levels. **(a)** Potentiation is associated with an increase in the number of pre-synaptic vesicles, docked vesicles, and Active Zone area at the pre-synaptic level. Post-synaptically, potentiation is accompanied by enlargement of spine head volume, PSD and Axon Spine Interface. The opposite occurs with depotentiation. **(b)** The Active Zone area, the number of pre-synaptic voltage-dependent calcium channels and RIM proteins, and the number of post-synaptic AMPA receptors increase or decrease with potentiation and depotentiation, respectively. **(c)** Synaptic potentiation can lead to increased pre-synaptic Calcium influx and/or to larger variations of the postsynaptic membrane potential. Pre-synaptically, the total number of pre-synaptic vesicles and the number of docked vesicles correlate with the Active zone area, which directly correlate with the number of pre-synaptic voltage-dependent calcium channels and RIM proteins that control pre-synaptic Calcium influx and neurotransmitter probability release at a specific active zones [56]. Post-synaptically, ASI area, PSD area and spine head volume directly and strongly correlate with the number of AMPA receptors and with the amplitude of the excitatory post-synaptic potential. Pre and post-synaptic morphological markers of synaptic strength also linearly and positively correlate [42, 43, 44, 45]. Abbreviations: PSD, post-synaptic density, Cav2.1, voltage-dependent Calcium channel P/Q type; RIM1/2, Rab interacting molecule; AMPA, α-amino-3-hydroxy-5-methyl-4-isoxazolepropionic acid; EPSP, excitatory post-synaptic potential.; ΔF/F0, fold change in fluorescent signal relative to baseline.

Moreover, combined electrophysiological and morphological studies demonstrated that circuit rewiring and synaptic plasticity are not necessarily associated with formation or elimination of dendritic spines. For instance, while in young rat hippocampus, long-term potentiation (LTP) produces new dendritic spines [39], in the adult, new spine outgrowth is stalled in favour of synapse enlargement [35,40]. Synaptic strength (i.e. the magnitude of the post-synaptic response) is determined by the neurotransmitter release probability and by the conductance and number of excitatory post-synaptic receptors. Synaptic strength tightly correlates with synaptic morphological features, which can then be used to predict the amplitude of the post-synaptic response (Figure 2). Spine head volume and post-synaptic density (PSD) area are considered the best morphological markers of synaptic strength because they are positively and linearly correlated with each other [41] and with the amplitude of the post-synaptic currents AMPA-receptor mediated [42]. Spine head volume and PSD enlarge after synaptic long-term potentiation and shrink after depotentiation [43]. Spine head volume and PSD area are also positively correlated with the area of contact between the axon and the spine (axon-spine interface or ASI) [44], the number of pre-synaptic vesicles [41], docked vesicles [45], and post-synaptic glutamate receptors [46] (Figure 2). Computational models showed that the spine surface to volume ratio has an important effect on spatial and temporal calcium dynamics within the spine and hence on synaptic function [35,47]. Spine surface to volume ratio is dependent on both spine size and shape, as well as on the presence and size of organelles within the spine such as the spine apparatus and the smooth-endoplasmic reticulum (SER). Therefore, in addition to spine density, actual synapse density and strength must be considered to determine the nature of sleep-dependent synaptic plasticity.

## Recent ultrastructural data on sleep-dependent synaptic downscaling

**Figure 3:**
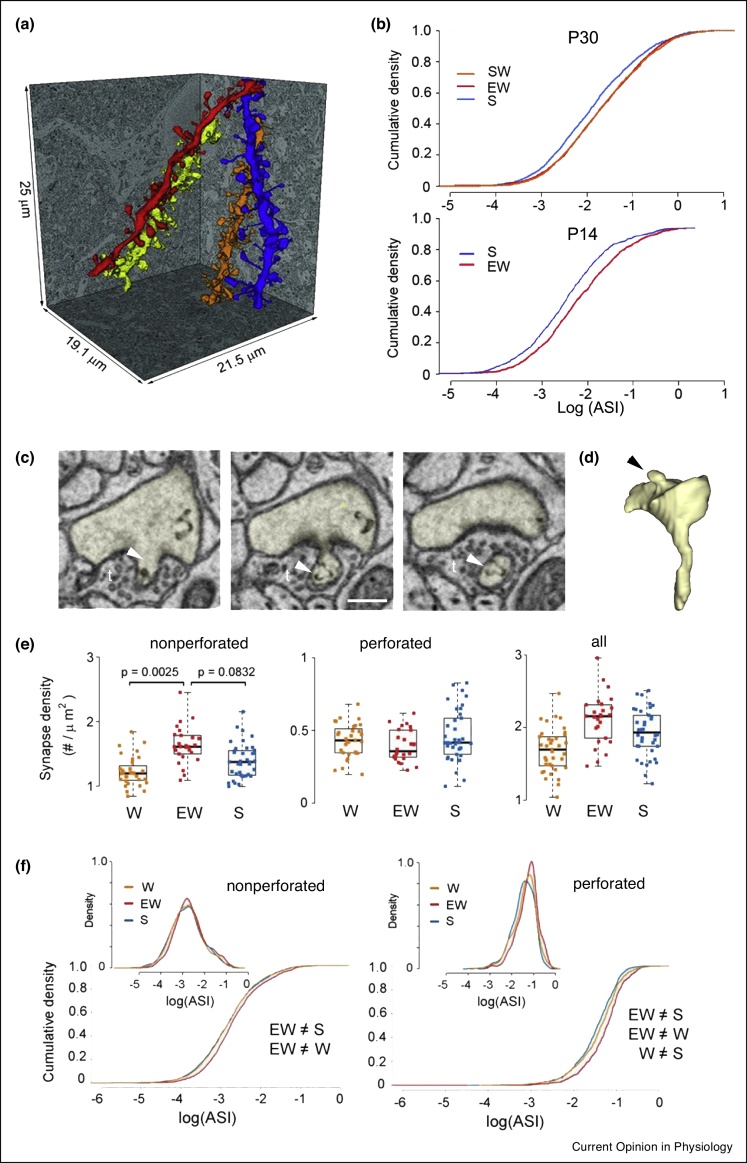
Ultrastructural synaptic plasticity across sleep and wake. **(a)** Reconstruction of four spiny dendritic segments sampled in primary somatosensory cortex from a volume of tissue imaged with serial block-face electron microscopy (SBF-SEM). **(b)** Top, ASI size in P30 mouse primary motor and somatosensory cortex declines in sleep by 18% according to a scaling relationship. Bottom, in P14 pups, ASI size in motor cortex scales down in sleep by 30%. **(c)** Three consecutive electron micrographs showing an example of a spine head (yellow) with a *spinula* (arrowhead) protruding into the pre-synaptic terminal (t), sampled from hippocampal CA1 stratum radiatum, scale bar = 300 nm. **(d)** 3d reconstruction of the spine and *spinula* (arrowhead) showed in (c). **(e)** Hippocampal synapse density (N of synapses per dendrite surface area) in each experimental group, shown for nonperforated, perforated, and all synapses. Each dot is one dendrite. **(f)** Distribution and cumulative plots of ASI sizes in each group, on a log scale, shown for hippocampal nonperforated and perforated synapses. Abbreviations: W, spontaneous wake group; S, sleep group; EW, enforced wake group; ASI, axon spine interface. (a) and (b) modified from Ref. [48] and [52]. (e) and (f) modified from Ref. [49].

The development of 3-dimensional electron microscopy techniques has allowed the reconstruction in 3 dimensions of cortical and hippocampal excitatory synapses across sleep and wake. In a first study, 30-day old (P30) mice were sacrificed after spending most of the previous 6−8 hours asleep, spontaneously awake or forcedly awake and about 8400 dendritic spines were reconstructed from random dendrites in layer II of the primary motor and somatosensory cortices [48^••^]. On average, 13% of all spines (range from 5% to up to 24%) were classified as ‘non-synaptic spines’ because they either lacked a clear post-synaptic density, a synaptic cleft or pre-synaptic vesicles in the structures surrounding the spine. Among the spines with a synapse, the ASIs shrank by 18% in the group of mice that spent most of the last 6−8 hours asleep compared to the mice that were awake, independently of time of day, suggesting that sleep promotes overall synaptic depotentiation (Figure 3a,b). Neither the abundance of ‘non-synaptic spines’ nor that one of those carrying a synapse changed significantly across the sleep-wake cycle. The sleep-dependent reduction in ASI area was not homogeneous: only small and medium size ASIs, that constituted about 80% of the population, downscaled after sleep, that is, shrank in a multiplicative manner. The population of biggest ASIs instead did not show downscaling. Moreover, ASIs that lacked non-SER recycling vesicles, tubules and multivesicular bodies in the spine head or neck (about 30–40% of all spines) were less likely to show sleep-dependent downscaling. Surprisingly, neither the presence of a spine apparatus (in about 30% of the spines) nor a pre-synaptic mitochondrion (found in about 32% of the terminals or boutons making synapse on the spines) influenced the likelihood of undergoing sleep-dependent downscaling.

Similarly, in the hippocampus, CA1 stratum radiatum, more than 7300 ASI were analysed after sleep, wake and enforced wake [49^••^] (Figure 3c–f). Consistently with previous reports, in CA1 two populations of synapses were found: perforated and non-perforated [50,51]. ASI were classified as perforated (22% of spine sample) according to the presence of a perforated PSD, a spinula (i.e. a protrusion of the post-synaptic element into the pre-synapse), and/or a spine apparatus in the spine. Whilst non-perforated ASI did not change across sleep and spontaneous wake, their density and size increased after enforced wake (Figure 3e,f). Perforated ASIs instead showed downscaling after sleep and upscaling after enforced wake relative to spontaneous wake, but without changing their density.

Finally, a recent study mapped the effect of sleep earlier in the development [52^•^]. Fourteen-day old (P14) mouse pups were either let sleep *ad libitum* for 4.5–6 hours or kept awake as much as possible with novel objects and gentle handling for the same time interval before brain collection. At this age, on average, spine density in primary motor cortex was similar to that measured in P30 mice but ASIs were smaller than at P30 (ASI in μm^2^, mean ± SD: P14, Sleep = 0.127 ± 0.141; P14 Enforced Wake = 0.168 ± 0.177; P30 sleep = 0.256 ± 0.289; P30 Enforced wake = 0.294 ± 0.324) and there were more non-synaptic spines (∼27% at P14 versus ∼13% at P30, no change between sleep and wake). As in P30 mice, in P14 pups, synaptic density did not change significantly across sleep and wake in motor cortex, and ASIs were smaller after sleep relative to enforced wake (Figure 3b). However, after sleep, the ASI population was homogenously downscaled by ∼30%, independently of ASI size and of the presence of specific organelles in the spine or in the pre-synaptic element.

## Conclusions

Detailed analysis of ultrastructural synaptic parameters is necessary to appreciate changes in synaptic strength that do not manifest necessarily as addition or elimination of new spines. 3d electron microscopy has been used to show that in the cerebral cortex of P30 mice, small and medium synapses underwent downscaling during sleep, whereas the largest synapses were protected. Since there is evidence that synapse size is positively correlated to synapse strength, by downscaling preferentially small and medium synapses and sparing the large ones, sleep would contribute to enhance the cortex signal to noise ratio and consolidate the synapses most potentiated during wake [9]. Experiments of longitudinal two-photon imaging suggest that spine size also correlates with spine stability [53^•^], with large spines being more stable than thin ones and contributing the most to the stability of long-term memories [54]. Since at P14, cortical synapses are smaller than at P30 and are all equally downscaled during sleep, it is likely that, at this age, synapses are highly plastic and not yet fully committed to long-term memory. However, the ultrastructural studies presented here were limited to small brain regions, excitatory synapses impinging on pyramidal neurons and were all conducted in P30 and P14 mice, leaving unresolved the question whether the same effects are also observed in other types of synapses and at different ages. They also highlighted that sleep-dependent synaptic plasticity can be heterogeneous in the brain and do not rule out that potentiation can also occur during sleep at specific synapses. Moreover, these studies did not establish whether the ultrastructural synaptic changes observed after sleep are directly linked to synaptic plasticity and learning occurred during wake rather than to overall changes in neuromodulator and hormonal levels across different physiological states. Another unanswered question is to what extent synaptic downscaling is necessary or sufficient for the cognitive improvement promoted by sleep. To this aims, examining synapse ultrastructure after learning of a sleep-dependent task or blocking synapse downscaling and assessing the effect on performance could be useful experiments. Future experiments should also aim to capture synaptic plasticity during sleep at the global and single synapse level. In this regard, by using two photon *in vivo* microscopy combined with fluorescent tagging of synaptic components (such as SEP-GluA1) one could reveal how single synapses behave across multiple sleep/wake cycles, across different periods of development and in relation to learning during waking experience. Multiplex electron microscopy [55] could help exploring how the identity of the pre-synaptic and post-synaptic elements as well as local differences in neuromodulator connectivity influence synaptic ultrastructure and plasticity during sleep. Moreover, novel algorithms of automatic image segmentation will speed up synapse tracing and will allow to extend the morphological investigation to larger brain areas.

## Funding

This work was supported by the Wellcome trust to MB (215267/Z/19/Z, 2019) and LdV (217546/Z/19/Z, 2019).

## Conflict of interest statement

Nothing declared.

## References and recommended reading

Papers of particular interest, published within the period of review, have been highlighted as:

• of special interest
•• of outstanding interest
